# Perspective for Aquaponic Systems: “*Omic*” Technologies for Microbial Community Analysis

**DOI:** 10.1155/2015/480386

**Published:** 2015-10-05

**Authors:** Perla Munguia-Fragozo, Oscar Alatorre-Jacome, Enrique Rico-Garcia, Irineo Torres-Pacheco, Andres Cruz-Hernandez, Rosalia V. Ocampo-Velazquez, Juan F. Garcia-Trejo, Ramon G. Guevara-Gonzalez

**Affiliations:** C. A. Ingeniería de Biosistemas, División de Investigación y Posgrado de la Facultad de Ingeniería, Universidad Autónoma de Querétaro, Centro Universitario, Cerro de las Campanas s/n, Colonia Las Campanas, 76010 Santiago de Querétaro, QRO, Mexico

## Abstract

Aquaponics is the combined production of aquaculture and hydroponics, connected by a water recirculation system. In this productive system, the microbial community is responsible for carrying out the nutrient dynamics between the components. The nutrimental transformations mainly consist in the transformation of chemical species from toxic compounds into available nutrients. In this particular field, the microbial research, the “*Omic*” technologies will allow a broader scope of studies about a current microbial profile inside aquaponics community, even in those species that currently are unculturable. This approach can also be useful to understand complex interactions of living components in the system. Until now, the analog studies were made to set up the microbial characterization on recirculation aquaculture systems (RAS). However, microbial community composition of aquaponics is still unknown. “*Omic*” technologies like metagenomic can help to reveal taxonomic diversity. The perspectives are also to begin the first attempts to sketch the functional diversity inside aquaponic systems and its ecological relationships. The knowledge of the emergent properties inside the microbial community, as well as the understanding of the biosynthesis pathways, can derive in future biotechnological applications. Thus, the aim of this review is to show potential applications of current “*Omic*” tools to characterize the microbial community in aquaponic systems.

## 1. Introduction

The continuous rise in global human population makes the expansion and intensification of our current food production systems necessary. In addition, in order to mitigate negative environmental impacts, it is also desirable to design new productive models with the capability to save energy cost, to reduce greenhouse gas emissions, to minimize waste disposals, and, even more, to recycle nutrients inside the system. From this approach, traditional food production systems have been under public eyes being questioned about its sustainability [1, 2]. One example is the case of aquaculture industry. Like other human activities, its problems concern the scientific community in many ways, but principally for its large waste discharges into environment and its accelerative growing rate [3]. However, as a result of the continuous innovations in the field, it has been possible to develop economically feasible systems capable to cultivate species at high densities, even with unfavorable climatic regime and limited water availability [3, 4]. These kinds of proposals are nowadays considered as culture models for sustainable food production systems [5].

Recirculation aquaculture systems (RAS) have been developed due to environmental restrictions in many countries with land and water limitations. RAS allows a reduction of water consumption due to waste management and nutrient recycling [3]. Historically, the concept of practical and efficient food production systems is not new. Cultures of China, Perú, and México had integrative systems which produce aquatic species and vegetables near to region of consumption [6]. In the XX century, the first attempts to create practical, efficient, and integrative fish production systems alongside vegetables were made in the 70s with the work of Lewis and Naegel [7–9]. These systems are known with the term “aquaponics.”

Aquaponics are a type of RAS in which water filtration technologies allow reuse of water for fish aquatic species production with integration of hydroponics [10]. The final byproduct of fish protein metabolism is ammonia (NH_3_) [11]. Ammonia accumulates in aquaculture ponds and it can be dangerous to fish at specific temperature and pH levels (above 30°, pH > 8.5) [12]. Wastes of ammoniacal nitrogen are transformed into less harmful compounds like nitrate by biological filtration [13, 14]. Accumulation of nitrate in water is less toxic for fish, but in RAS it is common to add make-up water in order to dissolve this compound (10% of total volume per day are make-up water) [15]. In contrast, aquaponics do not require water replacement; addition of make-up water is for losses because evaporation or replacement volume is less than 2% per day [16, 17].

The water is the common media that contain enough nitrogen compounds like ammonia, nitrate, nitrite, and other dissolved nutrients like phosphorus, potassium, and some other elements [18, 19]. These nutrients are enough for vegetable consumption [20]. Then, dissolved nutrients in the media are absorbed by root plants, optimizing the use of nutrients and water, and reduce wastes for fish and environmental impact [19]. On this way, the system allows minimizing resources as land, water, and energy [15].

One challenge of these systems is maintenance of water quality for both aquatic species and plants. For maintaince of water quality RAS have been utilized for solid removal and biological filtration. For this purpose, there are two or more components before the water returns to aquaculture pond [21]. In aquaponics, both solids removal and biological filtration are in the same component. Accumulation of uneaten food, fecal matter, and organic and nitrogen compounds in biofilter provide the adequate environment for microbial development [22]. However, the need of different physicochemical conditions in water for living components makes the management of the system very complex. The recommended pH for aquaculture systems is 6.5–8.5, for hydroponics is 5.5–6.5, and for nitrification process is 8.5. The pH is a parameter that can limit the development for plants, fish, or bacteria [14, 23]. Populations of microorganisms or microbial community in biofilter of aquaponics have an essential role in aquaponic systems development [21]. Biofiltration takes advantage of bacterial metabolic process. This process, the nitrification, is carried out in two steps, ammonia oxidizing and nitrate oxidizing. Each reaction involved different species of bacteria:* Nitrosomonas* and* Nitrobacter* [10, 24].

Probably best studied group of environmental importance in this type of ecosystems are nitrifying prokaryotes including both the ammonium oxidizing and the nitrite oxidizing prokaryotes [25]. However, microbial community in aquaponic system is not characterized. Due to biological interactions in biofilter of aquaponic system, microbial communities are very interesting to analyze.

The most important revolution in microbial ecology was the use of molecular techniques and DNA sequencing in phylogenetic studies and their applications to uncultured organisms [26]. These strategies can help to understand the interaction of microbial populations with each other and their environment as a consequence of nutrient input (from fish wastes) [16]. Moreover, using these tools, the vast prokaryotic diversity must be more revealed than with traditional techniques. Metagenomic techniques combined with next generations sequencing (NGS) and bioinformatic tools have boosted microbial ecology. The use of metagenomics approaches has allowed the discovery of large array of genes [26]. This modern approach allows knowledge of the diversity of metabolic functionality in order to understand in more detail the response of community at internal and external perturbations in relation to environmental dynamics and emergent properties [27]. With these studies it is possible to evaluate the potential of aquaponic microbial community for future biotechnological uses. The aim of this review is to show potential applications of current “*Omic*” and bioinformatic tools to characterize the microbial community of aquaponic systems.

## 2. Microbial Community in RAS

In RAS environment, aquaponic system is very important microbial community in the same order of magnitude as fish because they are directly involved into fish activities and their effect on water quality. The system provides different microniches for the microbial populations according to a differential gradient of oxygen and nutrients. Every microniche supports development of specific microbial populations [28]. Additionally freshwater, brackish, or marine RAS presented differences on microbial diversity [24, 29]. Biofilter component presents the most abundant content of microbes [22].

Microbial populations contribute to the processing of particulate and dissolved wastes of aquatic species (ammonia excreted by fish, and carbon and nitrogen accumulated from uneaten food and fecal matter). One of the most important conversions is carried out by nitrifying bacteria; they are involved in nitrification, ammonification, nitrate reduction, and denitrification processes [16, 22, 30]. Other microbial metabolisms are involved in proteolysis and sulfate reduction [30]. The populations are distributed according to respiratory metabolism determined in strict aerobic or microaerophilic and facultative anaerobes/aerobes, according to type of growth in fixed film bacteria or suspended bacterial, and according to the component of the system [22]. In general, the most common approach for nitrogen removal from water is based on the processes of aerobic autotrophic nitrification and anaerobic heterotrophic nitrification [31].

Autotrophic and heterotrophic microorganisms are present in RAS. Autotrophic organisms use CO_2_ as carbon source and inorganic nitrogen, sulphur, or iron compounds as energy source. Plants, algae, and some bacteria in aquaponic systems present this metabolism. Heterotrophic organisms use carbohydrates, amino acids, peptides, and lipids as carbon and energy source. In the system, organic matter from uneaten feeds, excreta of aquatic species, and detritus are mineralized by this type of microorganisms [22, 32].

Autotrophic nitrification removes ammonia at sufficient rate to maintain water quality at a level to prevent ammonia toxicity to the fish [33]. However, autotrophs are vulnerable to high loads of ammonium and organic matter. To overcome this latter situation, ammonia removal is in a very low level of removal, then making more components on the system for optimal ammonium removal necessary and then creating the need of additional steps in nitrogen oxidizing [34].

On the other hand, heterotrophic bacteria constitute an important factor in terms of O_2_ consumption and compete with autotrophic bacteria, diseases in fish and later in human. Some populations of these bacteria are suspected of having a positive effect against pathogenic bacteria [33]. Heterotrophic microorganisms exhibit higher growth rates than autotrophs and can use organic substrates as source of carbon and energy to convert ammonium into nitrogenous gas under aerobic conditions (heterotrophic nitrification) [31, 34]. The main source of heterotrophic bacteria is within the biofilter. Bacteria of heterotrophic nitrification are probably ideal prokaryotes for coupled nitrification-denitrification in wastewater treatment and, probably, the most abundant microorganisms in aquaponic systems [25]. The dissolved organic carbon (C) accumulated is the main source of C for heterotrophic bacteria. High concentration of organic carbon affects negatively nitrate production; it means concentration of nitrite was always very low [28, 35]. Some strains of heterotrophic nitrifiers had the capability to use nitrite (NO_2_) and nitrate (NO_3_) as the source for nitrogen for growth and as an energy source for denitrification [36].

**Figure 1:**
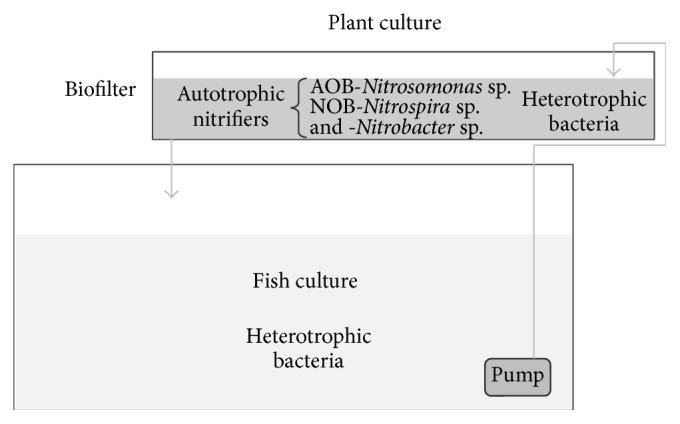
General distribution of microbial populations in aquaponic systems.

Ammonia Oxidizer Bacteria (AOB), like* Nitrosococcus*,* Nitrosospira*, and* Nitrosomonas* oxidized ammonia to nitrite. The general microdistribution of nitrifiers is that AOB live in dense clusters and their occurrence is reasonably well-correlated with oxygen content. These bacteria depend on availability of ammonia as their sole source of energy. On the other hand, Nitrite Oxidizer Bacteria (NOB) oxidized nitrite to nitrate by some* Nitrospira* sp. and* Nitrobacter*. These bacteria integrate more open aggregations but may also be found distributed in the biofilm systems. Another general observation is that* Nitrospira* spp., the dominant NOB in most systems, can still be detected below the oxic-anoxic interface, although in lower numbers and using small amounts of nitrite, and, in comparison with* Nitrobacter*, use oxygen more efficiently [22, 25]. The aforementioned theoretical distribution of autotrophic and heterotrophic bacteria in aquaponic systems is showed in Figure 1. The heterotrophic bacteria will be distributed near to outlet of flux water pumped from fish pond due to higher concentration of nutrients and inside the pond culture near to sediment. Autotrophic bacteria like strains of AOB-*Nitrosomonas* sp. will be in clusters in the middle of biofilter (here nutrient concentrations like ammonium and organic matter are lower) but in a portion of high O_2_ concentration; meanwhile NOB-*Nitrobacter* sp. and -*Nitrospira* will be in open aggregations in a portion of the oxic-anoxic interface in the middle of biofilter.

During oxidation of NH_4_^+^, pH increased from 7.1 to 8.45 under high ammonium loads. Ammonia Oxidizers Bacteria (AOB) and Nitrite Oxidizers Bacteria (NOB) are inhibited by free ammonia in range from 10 to 150 mg/L and from 0.1 to 1.0 mg/L, respectively. Free ammonia is NH_3_, the toxic form of ammoniacal N. High free ammonia (NH_3_) might inhibit the heterotrophic nitrification activity but not the growth. Heterotrophic nitrification and cellular growth differ according to pH conditions. Highest removal of ammonium (54.7%) and oxygen demand was presented at 7.5 pH (±0.5). At lower pH values or at more alkalinity, the growth of heterotrophic bacteria of group* Acinetobacter* increased. Efficient removal of ammonium at the slightly alkaline environment may be caused by more free ammonia contained in medium, which is preferentially by ammonia monooxygenase (*amoA*) [34].

High ratio of C/N helps to maintain safety values of toxic ammonium inside the system, mainly by its utilization on prokaryotic cell synthesis processes. There is evidence that intracellular nitrogen concentration removed from NH_4_^+^-N has close values from 52% to 56%. It means that bacterial growth was preferentially proceeding at high C/N ratios [28, 34].

### 2.1. Microbial Diversity Characterization

In 2000 decade, some species have been characterized in diverse components of RAS and mainly on biofilters [30, 37–39]. Considering studies of microbial populations that can be cultured, most of fixed bacteria were found in biofilter. Average CFU in biological filter was 7.3 × 10^6^  ± 7.25 × 10^6^ g^−1^ of media. Bacterial density in the inlet of biofilter was in lower level than in the outlet. Concentration of bacteria on the biofilter media was 5.1 ± 3.43 × 10^6^ to 1.1 × 10^8^  ± 3.41 × 10^7^. Thus, bacterial concentration does not depend of fish stocking density [28].

Several studies have been done in order to characterize microbial communities in RAS with freshwater. These studies revealed that the main bacterial groups presented in freshwater RAS biofilter were Actinobacteria, *α*-proteobacteria, *β*-proteobacteria, *γ*-proteobacteria, Bacilli, Bacteroidetes, Nitrospirae, Planctomycetes, and Sphingobacteria and the genus* Nitrosomonas* (Table 1). From these bacterial groups only* Hyphomicrobium facilis*,* Rhizobium* sp.,* Flavobacterium* sp.,* Sphingobacterium* sp.,* Comamonas* sp.,* Rhodobacter* sp.,* Acinetobacter* sp.,* Aeromonas* sp.,* Pseudomonas* sp.,* Flexibacter* sp.,* Pirellula staleyi*,* Nitrospira moscoviensis*, and* Nitrosomonas oligotropha* are common genera in systems with high richness and diversity.

PCR-based molecular techniques have mainly been used to describe microbial diversity using denaturing gradient gel electrophoresis (DGGE), microscopy using FISH (fluorescence* in situ* hybridization), and/or cloning 16S rRNA gene fragments [25, 30, 39, 42]. The last molecular technique is the most common for study of microbial communities in RAS with freshwater. For AOB, comparison between phylogenies based on 16S rRNA genes was done with* amoA* (gene of active subunit of monooxygenase),* nirK* (nitrite reductase gen), and* norB* (nitric oxide reductase) [25, 43].

The analysis using 16S rRNA genes as a phylogenetic marker was a revolutionary strategy for microbial ecology with cultured-independent method being developed since 90s, after the work of Lane and collaborators [44]. The 16S rRNA gene in bacteria contains highly conserved and variable interspersed regions that allow a reliable and detailed microbial classification. For this molecular technique the correct selection of primers is critical. Some pairs of primers can overestimate or underestimate species richness; it implied uncertain biological conclusions. This happened when primers selected do not anneal equally to DNA target in all members of community and the amplification was carried out on certain taxonomic group [45]. Some particular regions are recommended to obtain representational characterization in complex microbial community [45, 46].

Differences in microbial communities represent their unique and complex environments [16]. Microbial communities in aquatic system or in RAS are as complex as changes in environmental variables according to period of time [30, 39, 47]. Besides, every aquatic species in a RAS introduces its own unique microbial flora [30]. Aquaponic RAS system introduces additional living component compared to other RAS analyzed. Plants can introduce their own microbial flora to the system, thus making the study of the changes on microbial diversity very interesting. Ammonia Oxidizing Bacteria (AOB)* Nitrosomonas communis* introduced in rhizoplane of aquaponic plants has been isolated and identified [48]. Other processes of reduction/uptake of nitrogen compounds are carried out by eukaryotic microorganisms like diatoms, algae, and fungi [49]. Less well-studied is the heterotrophic nitrification carried out by fungi. These organisms have been associated with assimilatory nitrate reduction in RAS, removed ammonium, and nitrite and protein [49, 50]. These eukaryotic microorganisms have an important pathogen relationship with higher plants in humidity environments. Nowadays, there are no works reported about an analysis of bacterial or eukaryotic community in aquaponic systems. The microbial characterization on this field has been done in order to determine the presence of bacterial pathogens for human and for aquatic species [5, 51, 52].

## 3. Pathogens in Aquaponic Systems

Aquaponic systems have been used as sustainable agricultural systems [5, 51]. With the same volume of water for fish production can be produced edible vegetables. These systems are discussed as regards their utilization in improving sustainability through management and integration of the living components [10]. Many species of bacteria and coliforms are inherently present in aquaponic recirculating biofilter carrying out transformations of organic matter and wastes of fishes. This implies the presence of many microorganisms that can be pathogens for plants, fishes, and, mostly, human.

One of the most important considerations for this food production system is food safety. In agricultural systems, the evaluation of food safety is emerging as a critical procedure in harvesting and management operations. For this purpose, some microorganisms have been considered as safety-indicators for products and water quality in the system [5]. Some of these safety-indicators are* Escherichia coli* and* Salmonella* spp. These microorganisms are typically found in the intestines of warm-blooded animals like birds, mice, cattle, and others. They are common indicators of fecal contamination and microbial water quality. These bacteria are zoonotic enteric bacteria transient in fish gut microflora from contaminated water in open systems because of animals like birds [29]. Research on aquaponic fields has been carried out recently in order to ascertain microbial safety of its by-products [5, 51]. The microbial profile of lettuce produced under soil-free (aquaponics)* versus* in-soil has been evaluated. Comparative analysis showed significant differences between aquaponic and conventional lettuce in aerobic plate counts (APC), coliform,* E. coli*, and yeast count. Aquaponics had significantly lower concentration of coliform (no detectable* E. coli* were observed), spoilage and fecal microorganisms (lettuce from market contained 2–3.5 log CFU* E. coli*/g), and yeast counts (2-3 log CFU yeast/g for aquaponic and 5.5–5 log CFU yeast/g for conventional and organic lettuce). The later work suggests postharvest contamination due to packaging process and transport that conventional and organic lettuce suffered from in contrast to aquaponic one, in which the postharvest process was minimum [51].

Other works evaluated microbial water quality related to food safety in aquaponic system. This report analyzed plant and fish tissue, water, and supplement aquaponic input samples (that can be a contamination vector) from 11 different farms in Hawaii for approximately one year. Methodology used for food safety determination was the traditional microbial isolation of* E. coli* O157:H7 and* Salmonella*. The results showed very low levels of* E. coli* during initial sampling period according to EPA standards for recreational use of water. Plant and fish tissue analyzed and supplement inputs were shown to have very low levels of generic* E. coli* or undetectable* E. coli* O157:H7 and* Salmonella* [5]. Aforementioned works analyzed microbial profile of only two bacteria related with pathogenicity in humans. However microbial determination was carried out with conventional methods for microbial detection. This can be likely conducted to analyze a short range of microbial pathogens, because fish and plants pathogens were not considered in the study. For a deep microbial profile the use of modern metagenomic approaches is necessary.

On the other hand, some pathogens in biofilter component in RAS have been identified by 16S RNA clone library and DGGE (Table 1). Some strains of* Bacillus* sp. (like* B. mycoides*),* Aeromonas* sp.,* Acinetobacter* sp.,* Pseudomonas* sp.,* Edwardsiella* sp.,* Comamonas* sp., and* Flavobacterium* sp. are related with pathogenicity in fish [37]. Other pathogens found in biofilters are related to fish and human pathogenicity like* Vibrio*,* Erwinia*,* Coxiella*, and* Aeromonas* [16]. Species of* Vibrio* have been isolated from freshwater, estuarine, and seawater environments, although most of them are probably saprophytic [28].

Biosafety of aquaponic RAS will depend on correct management and control of opportunist microbial proliferation in the system [22]. Metagenomic and metatranscriptomic profile can be a powerful tool for determining the diversity of pathogens and functional activity that can help to understand their relationship with other microbes and possibly its regulation in the system. Metagenomics approaches allow the meta-analysis of diversity in microorganisms of the aquaponic environment [53–56].

## 4. “*Omic*” Tools for Future Analysis of the System

The development of sequencing and high-throughput methods for cloning microbial genes directly from environment has opened the possibilities for ecological microbiology, mostly considering that microbes possess the highest potential of producing bioactive metabolites, enzymes, and polymers and other tools with biotechnological application. The study of larger fragments of environmental DNA of whole community is known as environmental genomics, ecogenomics, or metagenomics [57]. The genetic, enzymatic, and metabolic pool is the result of a vast interaction cell-to-cell and/or synergistic or antagonistic relationships that could make the community perform as metaorganism with emergent properties [27].

### 4.1. Metagenomics for Microbial Diversity Description

PCR amplification of genes has allowed the study of microbial diversity. Throughout all the research done in this field the conclusion is that majority of prokaryotic diversity still remains unknown, mainly because these cells cannot be grown under laboratory conditions [58, 59]. Several works PCR-based molecular techniques for study of microbial diversity since about three decades ago have been carried out [60]. These tools has allowed to have a look of general scene of microbial diversity in environmental samples. However, techniques derived from PCR, like 16S rRNA, hybridization, and DGGE/TGGE, among others, have their limitations and only can give some information about communities [61]. The amplification of 16S rRNA gene technique is based on amplification of hypervariable regions of the gene anchoring to conserved sequences. There are nine (9) hypervariable regions named V1–V9 that spanned between 50 and 100 bp in length depending on region. Hypervariable regions are the key for universal microbial identification. Primers have been designed to amplify 16S rRNA hypervariable regions from large number of different bacteria species [26]. Primers that targeted regions V1–V3 and V7–V9 are recommended for obtaining representational characterization in complex microbial community [46]. The information of this technique indicates the taxonomic composition of the environmental sample [62]. There are several semiquantitative assays like FISH, MAR-FISH, and CARD-FISH* in situ* that identify prokaryotic cells without cultivation by applying fluorescence* in situ* hybridization (FISH) with ribosomal RNA (rRNA) targeted oligonucleotide probes. These oligonucleotides have an extention from 15 to 25 nucleotides in length and are labelled covalently at the 50' end with a fluorescent dye. After stringent washing, specifically stained cells are detected via epifluorescence microscopy or flow cytometry [63]. Quantitative analyses of the composition and dynamics of microbial communities are an integral component of microbial ecology. These techniques in combination with 16S rRNA have allowed real progress in some cases, especially in very simple ecosystems such as endosymbionts or extreme environments. The contribution of these techniques to a better understanding of functionality of ecosystems like microbial communities in ocean environment is discussed [56, 64]. On the other hand, NGS technologies have more throughput because they have 100 times more capacity of sequencing than Sanger method. These technologies sequenced DNA molecules massively in parallel in a flow cell. The sequencing is carried out in two forms, in a continuous real time or in a stepwise iterative process. In both types of processes each clonal template or single DNA molecule is sequenced and can be quantified among the total sequences generated [26]. Moreover, these modern technologies focus on sequencing of large fragments of DNA as entire genomes or plasmids instead of gene(s) or operons. For this process is necessary to fragment the total DNA in pieces up to 700 bp, in the case of shotgun the fragments are of 3 kb, 8 kb, and 40 kb [26, 65]. After this step further bioinformatical analysis is necessary in which these fragments are assembled in linear sequences that conform part of genome or total genome [65, 66]. The assembling overlaps the different fragments and thus rebuilds complete linear sequences of the genome, known as contigs. The build of entire genome is a little difficult but possible if the sequenced fragments cover the entire genome. The challenge of this strategy lies on computational effort that requires furthermore huge analysis and computational capacity [65, 67]. Metagenomics analysis comprises environmental single-gene surveys and random shotgun studies of all environmental genes. The former analysis focuses on metagenomic study by single targets amplified with PCR and, then, the products are sequenced. On the other hand, shotgun metagenomics is targeted in total DNA isolated from an environmental sample and then sequenced, resulting in a profile of all genes within the community. The basic definition of metagenomics is the analysis of genomic DNA from a whole community; this separates it from genomics, which is the analysis of genomic DNA from an individual organism or cell [68, 69]. Metagenomic studies combined with NGS technologies promise to be a tool that helps the evolution of microbial ecology at very fast step. Nowadays, there is a discussion on this topic because metagenomics allow microbial analysis on a low or medium complex ecosystem but in highly complex environment it has not been successful due to effort in heterogeneity assembly [26, 70].

### 4.2. Metatranscriptomics, Proteomics, and Metabolomics for Microbial Functionality Description

Metatranscriptomics, proteomics, and metabolomics can provide information of functional analyses in microbial community at different levels, gene expression, protein translation, and more recently the metabolite network, respectively.

Metatranscriptomic data are a set of cDNA derived from community RNA. This information can help to infer what are microorganisms doing in a precise moment, how is their reaction to the environment, and what are they saying to the neighboring cell and the community [71].

For transcriptomic approach the gene(s) is required isolated in precise time of expression. It shows that transcriptome was very different every time. The functional “*Omic*” study has two main objectives: determine differences in functionality and metabolic pool from each member of a community that produce different effects on the system and identify the variations within functional compositions of different communities [71].

The integration of metagenomic and metatranscriptomic data revealed many unassignable transcripts that make evident the gap in knowledge for gene-protein that enables the ecophysiology of microorganisms in the ecosystem [72]. The mRNA concentration has been used to approximate the concentration and activities of their corresponding proteins; however with recent technologies it has been demonstrated that concentration of transcripts helps to predict partially the protein abundance. The latter assumption suggests that there are other mechanisms of regulations influencing the level of proteins in cells [73].

On the other hand, proteomics is the analysis of proteome, the full complement of proteins expressed by an organism. The number of proteins in the proteome organism exceeds by far the number of genes [74]. Every fragment of DNA is biochemically similar to one another. However, every type of protein is very different to the others. This protein diversity represents one of the greatest challenges of “*Omic*” technologies because to define its own identity, quantity, structure, and functionality of complete complements of proteins and, moreover, to characterize how these properties change through every cellular context are very complex [75].

In contrast, metabolites are the end products of cellular regulatory processes that can be chemically transformed during metabolism and provide a functional state of cellular biochemistry. The level of these chemical entities can be regarded as the ultimate response of biological systems to genetic (posttranslational modifications) or environmental changes (epigenetic regulation). Metabolites serve as direct signatures of biochemical activity and therefore they are easy to correlate with phenotype making it a powerful tool in order to explode in different fields of science. In parallel with the terms “transcriptome” and “proteome,” the set of metabolites synthesized by a biological system constitute its “metabolome” [76]. This can be defined on all levels of complexity, such as organisms, tissue, cells, or cell compartments. For this reason in a biological experiment it is necessary to be specific about the environmental conditions as exactly as possible [77].

In metabolome analysis the most functional characterizations of genes involved in a metabolism are not based upon rigid biochemical testing. Many of putative function assignments of proteins do not describe biochemical function or biological role. It can be the result of gene duplication that is responsible for many enzyme isoforms and exhibits different characteristics. In contrast with transcriptome analysis (but in common with proteome analysis) methods are not available for amplification of metabolites and, therefore, sensitivity is a major issue. Metabolite products can be labile species and by their nature are chemically very diverse. For this reason, they are present in a wide dynamic range. On the other hand, in contrast with transcript or protein identification, metabolites are not organism specific and are not sequenced-dependent; thus when how to measure the metabolite once has been identified, the analytical protocol is equally applicable to prokaryotes, fungi, plants, and animals [78].

Biotechnology development is based on a very small diversity of species like* E. coli* and recent “*Omic*” tools offer high potential for discovery and exploitation of novel species, enzymes, and process that before were inaccessible [79, 80]. However, the data generated with these technologies have a small role on biotechnological research; most of novel developments occur on heterologous expression of enzymes. Other constrains with these approaches have been detected, that is, “under- or overestimation of the complexity of microbial diversity, limited data with the source of each sample, the identification of many genes, difficulties in integrating and comparing results obtained with different technologies, mismatched expectations between researchers who sought to generate understanding of ecological patterns with those who were excited to test the limits of new technology, and the lack of agreed upon data standards” [65, 79].

The experimental design and the adoption of minimum standards to generate an adequate number of samples that allows the significant statistical analysis are highly desirable for future “*Omic*” studies. This step can be the key for determining their patterns of cooccurrence on gene(s) with taxa that are difficult to characterize and dominant factors structuring the community across time and space [79].

There are many factors to take into account in experimental design: replicates that can consume time and cost, but it must be sufficient for biosystem description, the definition of the most significant source of variations in a given biosystem being difficult, choosing of sequencing platform (each one has differences in length of sequences needed and advantages and disadvantages), and interpretation of sequence data and metadata collection [79, 81].

The “*Omic*” technologies challenges for characterized microbial diversity are the experiment itself, the statistical analysis of results, and the biological interpretation, which is the most complex and time-consuming part.

## 5. Conclusions

“*Omic*” approaches as metagenomics and metatranscriptomics must be crucial in future studies of microbial diversity in aquaponic biosystems. In addition, other “*Omic*” approaches as proteomics and metabolomics, together with respective bioinformatic analysis, should increase the knowledge of the ecological role and functionality of microbial components in these study models.

## Figures and Tables

**Table 1:** Microorganisms identified in RAS biofilter component related with freshwater.

Group	Microorganism	Process	References
Actinobacteria	*Microbacterium imperiale *		[30]
	*Mycobacterium chitae *		[30]
	*Corynebacterium tuberculostearicum *	Pathogen in humans	[39]
	*Propionibacterium acnes *		[39]
Acidobacteria	*Acidobacteria bacterium *		[39]
Bacteroidetes	*Chryseobacterium* sp.	Some strains pathogen in humans	[37]
	*Flavobacteriales bacterium *	Sulfate reduction	[37]
	*Flavobacterium columnare *	Pathogen in fish	[39]
	*Flavobacterium* sp.	Heterotrophic denitrification	[38, 39]
	*Bacteroides plebeius *	Sulfate reduction	[39]
	*Myroides* sp.	Pathogen in humans	[37]
	*Sphingobacterium* sp.	Pathogen in fish	[30, 37, 39]
	*Flectobacillus *	Heterotrophic bacteria	[39]
α-Proteobacteria	*Agrobacterium tumefaciens *	Pathogen in superior plants/nitrogen fixation	[30]
	*Filomicrobium fusiforme *		[30]
	*Hyphomicrobium facilis *		[30, 39]
	*Hyphomicrobium denitrificans* sp.	Heterotrophic denitrification	[30]
	*Nitrobacter winogradskyi *	Nitrite oxidation	[30, 40]
	*Nordella oligomobilis *		[30]
	*Ochrobactrum anthropi *		[30]
	*Rhizobium* sp.	Nitrogen fixation	[30, 37, 39]
	*Rhodopseudomonas acidophila *		[30]
	*Rhodovulum euryhalinum *	Denitrification	[30]
	*Bradyrhizobium japonicum *		[39]
	*Woodsholea maritima *		[39]
	*Rhodobacter* sp.	Autotrophic denitrification/nitrogen fixation	[22, 30]
β-Proteobacteria	*Aquaspirillum* sp.	Pathogen in fish	[37]
	*Comamonas *	Heterotrophic denitrification/pathogen in fish	[22, 37, 39]
	*Azovibrio restrictus *		[30]
	*Thiobacillus thioparus *	Ammonia oxidation	[30]
	*Herbaspirillum* sp.		[39]
	*Ideonella dechloratans *	Heterotrophic bacteria	[39]
	*Rhodoferax fermentans *	Autotrophic denitrification	[30]
	*Nitrosomonas aestuarii *	Anammox	[38]
	*Nitrosomonas marina *	Anammox	[16]
	*Nitrosomonas oligotropha *	Anammox	[38]
γ-Proteobacteria	*Gemmatimonas aurantiaca *		[39]
	*Acinetobacter* sp.	Heterotrophic bacteria	[37, 39]
	*Aeromonas* sp.	Heterotrophic denitrification/pathogen in fish and humans	[37, 39]
	*Pseudomonas* sp.	Heterotrophic denitrification/pathogen in fish and humans	[16, 22, 37, 39]
	*Marinobacter* sp.		[39]
	*Vibrio* sp.		[39]
	*Edwardsiella* sp.	Pathogen in fish	[37]
ϵ-Proteobacteria	*Arcobacter nitrofigilis *	Nitrogen fixation	[39]
Firmicutes	*Bacillus* sp.	Pathogen in fish	[37]
	*Lactobacillus paraplantarum *		[30]
	*Lactococcus lactis *		[39]
	*Macrococcus brunensis *		[30]
	*Macrococcus lamae *		[30]
	*Sarcina* sp.	Dissimilatory nitrate reduction to ammonium (DNRA)	[37]
Sphingobacteria	*Flexibacter* sp.		[30, 39]
	*Runella slithyformis *		[39]
Verrucomicrobia	*Verrucomicrobia spinosum *		[39]
Planctomycetes	*Pirellula staleyi *	Anammox	[30, 41]
	*Planctomycetales* sp.	Anammox	[38]
	*Planctomyces maris *	Anammox	[38]
	*Planctomicetes* sp.	Nitrite oxidation	[38]
Nitrospirae	*Nitrospira moscoviensis *	Nitrite oxidation	[30, 38, 39]

All microorganisms of this table were analysed with 16S rRNA clone library method, denaturing gradient gel electrophoresis (DGGE), and few cases with biochemical procedures.

## References

[1] Beddington S. J. (2011). The future of food and farming. *International Journal of Agricultural Management*.

[2] Anastas P. T., Zimmerman J. B. (2003). Design through the 12 principles of green engineering. *Environmental Science and Technology*.

[3] Martins C. I. M., Eding E. H., Verdegem M. C. J. (2010). New developments in recirculating aquaculture systems in Europe: a perspective on environmental sustainability. *Aquacultural Engineering*.

[4] Bostock J., McAndrew B., Richards R. (2010). Aquaculture: global status and trends. *Philosophical Transactions of the Royal Society B*.

[5] Fox B. K., Tamaru C. S., Hollyer J. (2012). *A Preliminary Study of Microbial Water Quality Related to Food Safety in Recirculating Aquaponic Fish and Vegetable Production Systems*.

[6] Jones S. (2002). Evolution of aquaponics. *International Journal of Agricultural Management*.

[7] Lewis W. M., Wehr L. W. (1976). A fish-rearing system incorporating cages, water circulation, and sewage removal. *The Progressive Fish-Culturist*.

[8] Naegel L. C. A. (1977). Combined production of fish and plants in recirculating water. *Aquaculture*.

[9] Lewis W. M., Yopp J. H., Schramm H. L., Brandenburg A. M. (1978). Use of hydroponics to maintain quality of recirculated water in a fish culture system. *Transactions of the American Fisheries Society*.

[10] Tyson R. V., Treadwel D. D., Simonne E. H. (2011). Opportunities and challenges to sustainability in aquaponic systems. *HortTechnology*.

[11] Durborow R. M., Crosby D. M., Brunson M. W. (1997). Ammonia in fish ponds. *Journal of the Fisheries Research Board of Canada*.

[12] Alatorre-Jácome O., Rico-García E., García-Trejo F., Soto-Zarazúa G. M. (2011). *Aquaculture Water Quality for Small-Scale Producers*.

[13] Durborow R. M., Crosby D. M., Brunson M. W. (1997). *Nitrite in Fish Ponds*.

[14] Tyson R. V., Simonne E. H., White J. M., Lamb E. M. (2004). Reconciling water quality parameters impacting nitrification in aquaponics: the pH levels. *Proceedings of the Florida State Horticultural Society*.

[15] Timmons M. B., Ebeling J. M., Wheaton F. W., Summerfelt S. T., Vinci J. B. (2002). *Recirculating Aquaculture Systems*.

[16] Schreier H. J., Mirzoyan N., Saito K. (2010). Microbial diversity of biological filters in recirculating aquaculture systems. *Current Opinion in Biotechnology*.

[17] Rakocy J. E., Masser M. P., Losordo T. M. (2006). Recirculating aquaculture tank production systems: aquaponics-integrating fish and plant culture. *SRAC Publication No.*.

[18] Losordo T. M., Michael M. P., Rakocy J. (1998). Recirculating aquaculture tank production systems. an overview of critical considerations. *Publication*.

[19] Diver S.

[20] Yeo S. E., Binkowski F. P., Morris J. E. (2004). *Aquaculture Effluents and Waste by-Products: Characteristics, Potential Recovery, and Beneficial Reuse*.

[21] Bai Y., Zhang J., Li Y.-F., Gao Y.-N., Li Y. (2005). Biomass and microbial activity in a biofilter during backwashing. *Journal of Zhejiang University: Science B*.

[22] Rurangwa E., Verdegem M. C. (2015). Microorganisms in recirculating aquaculture systems and their management. *Reviews in Aquaculture*.

[23] Tyson R. V., Simonne E. H., Treadwell D. D., White J. M., Simonne A. (2008). Reconciling pH for ammonia biofiltration and cucumber yield in a recirculating aquaponic system with perlite biofilters. *HortScience*.

[24] Tal Y., Watts J. E. M., Schreier S. B., Sowers K. R., Schreier H. J. (2003). Characterization of the microbial community and nitrogen transformation processes associated with moving bed bioreactors in a closed recirculated mariculture system. *Aquaculture*.

[25] Revsbech N. P., Risgaard-Petersen N., Schramm A., Nielsen L. P. (2006). Nitrogen transformations in stratified aquatic microbial ecosystems. *Antonie van Leeuwenhoek*.

[26] Nikolaki S., Tsiamis G. (2013). Microbial diversity in the era of omic technologies. *BioMed Research International*.

[27] Konopka A. (2009). What is microbial community ecology?. *The ISME Journal*.

[28] Leonard N., Blancheton J. P., Guiraud J. P. (2000). Populations of heterotrophic bacteria in an experimental recirculating aquaculture system. *Aquacultural Engineering*.

[29] Sugita H., Shibuya K., Shimooka H., Deguchi Y. (1996). Antibacterial abilities of intestinal bacteria in freshwater cultured fish. *Aquaculture*.

[30] Sugita H., Nakamura H., Shimada T. (2005). Microbial communities associated with filter materials in recirculating aquaculture systems of freshwater fish. *Aquaculture*.

[31] Joo H.-S., Hirai M., Shoda M. (2005). Characteristics of ammonium removal by heterotrophic nitrification-aerobic denitrification by *Alcaligenes faecalis* no. 4. *Journal of Bioscience and Bioengineering*.

[32] Del Giorgio P. A., Cole J. J. (1998). Bacterial growth efficiency in natural aquatic systems. *Annual Review of Ecology and Systematics*.

[33] Michaud L., Blancheton J. P., Bruni V., Piedrahita R. (2006). Effect of particulate organic carbon on heterotrophic bacterial populations and nitrification efficiency in biological filters. *Aquacultural Engineering*.

[34] Ren Y.-X., Yang L., Liang X. (2014). The characteristics of a novel heterotrophic nitrifying and aerobic denitrifying bacterium, *Acinetobacter junii* YB. *Bioresource Technology*.

[35] Leonard N., Guiraud J. P., Gasset E., Cailleres J. P., Blancheton J. P. (2002). Bacteria and nutrients—nitrogen and carbon—in a recirculating system for sea bass production. *Aquacultural Engineering*.

[36] Padhi S. K., Tripathy S., Sen R., Mahapatra A. S., Mohanty S., Maiti N. K. (2013). Characterisation of heterotrophic nitrifying and aerobic denitrifying *Klebsiella pneumoniae* CF-S9 strain for bioremediation of wastewater. *International Biodeterioration & Biodegradation*.

[37] Schneider O., Chabrillon-Popelka M., Smidt H. (2007). HRT and nutrients affect bacterial communities grown on recirculation aquaculture system effluents. *FEMS Microbiology Ecology*.

[38] Itoi S., Niki A., Sugita H. (2006). Changes in microbial communities associated with the conditioning of filter material in recirculating aquaculture systems of the pufferfish *Takifugu rubripes*. *Aquaculture*.

[39] Itoi S., Ebihara N., Washio S., Sugita H. (2007). Nitrite-oxidizing bacteria, *Nitrospira*, distribution in the outer layer of the biofilm from filter materials of a recirculating water system for the goldfish *Carassius auratus*. *Aquaculture*.

[40] Hovanec T. A., DeLong E. F. (1996). Comparative analysis of nitrifying bacteria associated with freshwater and marine aquaria. *Applied and Environmental Microbiology*.

[41] Tal Y., Watts J. E. M., Schreier H. J. (2006). Anaerobic ammonium-oxidizing (Anammox) bacteria and associated activity in fixed-film biofilters of a marine recirculating aquaculture system. *Applied and Environmental Microbiology*.

[42] Kowalchuk G. A., Stephen J. R. (2001). Ammonia-oxidizing bacteria: a model for molecular microbial ecology. *Annual Review of Microbiology*.

[43] Cébron A., Garnier J. (2005). *Nitrobacter* and *Nitrospira* genera as representatives of nitrite-oxidizing bacteria: detection, quantification and growth along the lower Seine River (France). *Water Research*.

[44] Lane D. J., Pace B., Olsen G. J., Stahl D. A., Sogin M. L., Pace N. R. (1985). Rapid determination of 16S ribosomal RNA sequences for phylogenetic analyses. *Proceedings of the National Academy of Sciences of the United States of America*.

[45] Shah N., Tang H., Doak T. G., Ye Y. (2010). Comparing bacterial communities inferres from 16s rrna gene sequencing and shotgun metagenomics. *WSPC—Proceedings*.

[46] Kumar P. S., Brooker M. R., Dowd S. E., Camerlengo T. (2011). Target region selection is a critical determinant of community fingerprints generated by 16s pyrosequencing. *PLoS ONE*.

[47] Gilbert J. A., Steele J. A., Gregory Caporaso J. (2012). Defining seasonal marine microbial community dynamics. *ISME Journal*.

[48] Tokuyama T., Mine A., Kamiyama K. (2004). Nitrosomonas communis strain YNSRA, an ammonia-oxidizing bacterium, isolated from the reed rhizoplane in an aquaponics plant. *Journal of Bioscience and Bioengineering*.

[49] Yusoff F. M., Banerjee S., Khatoon H., Shariff M. (2011). Biological approaches in management of nitrogenous compounds in aquaculture systems. *Dynamic Biochemistry, Process Biotechnology & Molecular Biology*.

[50] van Rijn J., Tal Y., Schreier H. J. (2006). Denitrification in recirculating systems: theory and applications. *Aquacultural Engineering*.

[51] Sirsat S., Neal J. (2013). Microbial profile of soil-free versus in-soil grown lettuce and intervention methodologies to combat pathogen surrogates and spoilage microorganisms on lettuce. *Foods*.

[52] Martins P., Cleary D. F. R., Pires A. C. C. (2013). Molecular analysis of bacterial communities and detection of potential pathogens in a recirculating aquaculture system for *Scophthalmus maximus* and *Solea senegalensis*. *PLoS ONE*.

[53] Tamames J., Abellán J. J., Pignatelli M., Camacho A., Moya A. (2010). Environmental distribution of prokaryotic taxa. *BMC Microbiology*.

[54] Dinsdale E. A., Edwards R. A., Hall D. (2008). Functional metagenomic profiling of nine biomes. *Nature*.

[55] Breitbart M., Hoare A., Nitti A. (2009). Metagenomic and stable isotopic analyses of modern freshwater microbialites in Cuatro Ciénegas, Mexico. *Environmental Microbiology*.

[56] Parthasarathy H., Hill E., MacCallum C. (2007). Global ocean sampling collection. *PLoS Biology*.

[57] Rodríguez-Valera F. (2004). Environmental genomics, the big picture?. *FEMS Microbiology Letters*.

[58] Whitman W. B., Coleman D. C., Wiebe W. J. (1998). Prokaryotes: The unseen majority. *Proceedings of the National Academy of Sciences of the United States of America*.

[59] Handelsman J. (2004). Metagenomics: application of genomics to uncultured microorganisms. *Microbiology and Molecular Biology Reviews*.

[60] Hugenholtz P., Goebel B. M., Pace N. R. (1998). Impact of culture-independent studies on the emerging phylogenetic view of bacterial diversity. *Journal of Bacteriology*.

[61] Muyzer G. (1999). DGGE/TGGE a method for identifying genes from natural ecosystems. *Current Opinion in Microbiology*.

[62] Kemp P. F., Aller J. Y. (2004). Bacterial diversity in aquatic and other environments: what 16S rDNA libraries can tell us. *FEMS Microbiology Ecology*.

[63] Wagner M., Hornt M., Daims H. (2003). Fluorescence in situ hybridisation for the identification and characterisation of prokaryotes. *Current Opinion in Microbiology*.

[64] Gilbert J. A., Dupont C. L. (2011). Microbial metagenomics: beyond the genome. *Annual Review of Marine Science*.

[65] Bonilla-Rosso G., Eguiarte L. E., Souza V. (2008). Metagenómica, genómica y ecología molecular: La nueva ecología en el bicentenario de darwin. *TIP Revista Especializada en Ciencias Químico-Biológicas*.

[66] Bansal A. K. (2005). Bioinformatics in microbial biotechnology—a mini review. *Microbial Cell Factories*.

[67] Venter J. C., Remington K., Heidelberg J. F. (2004). Environmental genome shotgun sequencing of the sargasso sea. *Science*.

[68] Riesenfeld C. S., Schloss P. D., Handelsman J. (2004). Metagenomics: genomic analysis of microbial communities. *Annual Review of Genetics*.

[69] Kennedy J., Flemer B., Jackson S. A. (2010). Marine metagenomics: new tools for the study and exploitation of marine microbial metabolism. *Marine Drugs*.

[70] Schloss P. D., Handelsman J. (2003). Biotechnological prospects from metagenomics. *Current Opinion in Biotechnology*.

[71] Prakash T., Taylor T. D. (2012). Functional assignment of metagenomic data: challenges and applications. *Briefings in Bioinformatics*.

[72] Gilbert J. A., Margaret H., Kwon Y. M., Ricke S. R. (2011). Gene expression profiling: metatranscriptomics. *High-Throughput next Generation Sequencing*.

[73] Vogel C., Marcotte E. M. (2012). Insights into the regulation of protein abundance from proteomic and transcriptomic analyses. *Nature Reviews Genetics*.

[74] Graveley B. R. (2001). Alternative splicing: increasing diversity in the proteomic world. *Trends in Genetics*.

[75] Phizicky E., Bastiaens P. I. H., Zhu H., Snyder M., Fields S. (2003). Protein analysis on a proteomic scale. *Nature*.

[76] Patti G. J., Yanes O., Siuzdak G. (2012). Innovation: metabolomics: the apogee of the omics trilogy. *Nature Reviews Molecular Cell Biology*.

[77] Fiehn O. (2002). Metabolomics—the link between genotypes and phenotypes. *Plant Molecular Biology*.

[78] Hollywood K., Brison D. R., Goodacre R. (2006). Metabolomics: current technologies and future trends. *Proteomics*.

[79] Knight R., Jansson J., Field D. (2012). Unlocking the potential of metagenomics through replicated experimental design. *Nature Biotechnology*.

[80] Xu J. (2006). Microbial ecology in the age of genomics and metagenomics: concepts, tools, and recent advances. *Molecular Ecology*.

[81] Frey K. G., Herrera-Galeano J. E., Redden C. L. (2014). Comparison of three next-generation sequencing platforms for metagenomic sequencing and identification of pathogens in blood. *BMC Genomics*.

